# Comparison of the Luminal and Mucosa-Associated Microbiota in the Colon of Pigs with and without Swine Dysentery

**DOI:** 10.3389/fvets.2017.00139

**Published:** 2017-08-24

**Authors:** Eric R. Burrough, Bailey L. Arruda, Paul J. Plummer

**Affiliations:** ^1^Department of Veterinary Diagnostic and Production Animal Medicine, College of Veterinary Medicine, Iowa State University, Ames, IA, United States; ^2^Department of Veterinary Microbiology and Preventive Medicine, College of Veterinary Medicine, Iowa State University, Ames, IA, United States

**Keywords:** swine, microbial profiling, metagenomics, swine dysentery, *Brachyspira hyodysenteriae*, *Brachyspira hampsonii*

## Abstract

Colonic contents and mucosal scrapings from pigs inoculated with *Brachyspira hyodysenteriae* or *Brachyspira hampsonii* were collected at necropsy and classified as either positive (*n* = 29) or negative (*n* = 7) for swine dysentery (SD) based upon lesions and positive culture from the source pig. The microbiota in each sample was analyzed by bacterial census taking (16S rRNA gene sequencing). Procrustes analysis revealed similar clustering by disease classification with a relatively high M2 value (0.44) suggesting differences in the microbiota between mucosal and luminal samples from the same pig. In both sample types, differences in richness and beta diversity were observed between disease statuses (*P* ≤ 0.014). The relative abundance of *Brachyspirales, Campylobacterales, Desulfovibrionales*, and *Enterobacteriales* was higher in pigs with dysentery for both mucosal scrapings and luminal samples while *Clostridiales, Erysipelotrichales*, and *Fusobacteriales* were significantly more abundant in the luminal contents only. For inoculated pigs that did not develop dysentery, *Burkholderiales* were more abundant in both sample types, *Bacteroidales* and *Synergistales* were more abundant in mucosal scrapings, and *Lactobacillales* and *Bifidobacteriales* were more abundant in luminal contents when compared with diseased pigs. Linear discriminant analysis of effect size revealed *Brachyspira, Campylobacter, Mogibacterium*, and multiple *Desulfovibrio* spp. as differential features in mucosal scrapings from pigs with dysentery while *Lactobacillus* and a *Bifidobacterium* spp. were differential in pigs without disease. These differential features were not observed in luminal samples. In summary, microbial profiles in both sample types differ significantly between disease states; however, evaluation of the mucosal microbiome specifically may be of higher value in elucidating bacterial mechanisms underlying development of SD.

## Introduction

Swine dysentery (SD) is characterized by severe mucohemorrhagic diarrhea and is associated with infection by strongly beta-hemolytic strains of *Brachyspira hyodysenteriae, Brachyspira hampsonii*, and *Brachyspira suanatina* (1). While these spirochetes are required for disease expression, SD only develops in pigs when one or more specific anaerobes are present in the microbiota (2, 3). Additionally, dietary modification can significantly alter the colonic microbiota of pigs and thereby increase or decrease expression of SD (4–6) suggesting that there may be specific microbial profiles that are permissive and resistant to SD expression.

With the advent of affordable next generation sequencing technology, research studies exploring microbial community profiles associated with heath and disease have flourished. For enteric disease states, many studies have focused on profiling the luminal contents or feces as these sample types are relatively easy to obtain; however, changes observed in these samples likely represent an indirect measure at best of what is happening at the mucosal surface where bacteria interact more intimately with the host and induce disease. In the case of SD, where there are profound changes in the colonic mucosa and where the etiologic agent can be readily visualized microscopically within the mucus layer, crypts, goblet cells, and epithelium (7), it seems logical to explore the microbiome directly associated with this biological niche in efforts to identify potential microbial biomarkers associated with disease susceptibility.

Accordingly, the microbial profiles of colonic contents and mucosal scrapings from pigs inoculated with *B. hyodysenteriae* or *B. hampsonii* were compared to determine differences between the microbiota of those pigs that developed SD following inoculation and those that did not. The *a priori* hypothesis of this study was that the microbial profiles in mucosal scrapings and luminal contents from the same pig differ significantly and that mucosal scraping profiles may reveal potential biomarkers of resistance and susceptibility to development of SD.

## Materials and Methods

### Colonic Samples

Paired colonic content and mucosal scrapings from 36 commercial crossbred pigs receiving antibiotic-free rations and inoculated with either *B. hyodysenteriae* or *B. hampsonii* (5.8 × 10^5^–1.2 × 10^6^ CFU/ml once daily for 3 days) were collected at necropsy during a previous study (8). All animal procedures were approved by the Institutional Animal Care and Use Committee of Iowa State University (Log Number: 1-12-7283). Pigs were approximately 9 weeks old at the time of necropsy and were euthanized by barbiturate overdose within 72 h of SD development, which occurred between 6 and 14 DPI, or at the end of the study at 21 DPI. The spiral colon was exteriorized within approximately 5 min after death and luminal contents were collected from an incision at the apex of the spiral colon. A separate set of disinfected instruments was used for each pig and the luminal contents were collected into individual sterile 2.0 ml cryogenic vials (Corning Inc., Corning, NY, USA) and were snap frozen in liquid nitrogen. The apex of the spiral colon was then opened to reveal the mucosa, residual contents were gently removed from the surface, and a mucosal scraping was obtained using the blade of a post mortem knife. Mucosal scrapings were then transferred into individual sterile polystyrene snap-cap tubes (Corning Inc., Corning, NY, USA), refrigerated for 2–4 h, and then frozen and retained at −80°C until further processing for use in this study. The paired samples were classified as either positive (*n* = 29) or negative (*n* = 7) for SD based on the presence of mucohemorrhagic diarrhea, appropriate microscopic lesions in fixed colonic tissues (neutrophilic infiltration of the lamina propria and increased mucosal thickness), and recovery of a strongly hemolytic *Brachyspira* by selective anaerobic culture from the source pig. *Brachyspira* cultivation was performed anaerobically using selective agar containing spiramycin, rifampin, vancomycin, colistin, and spectinomycin with incubation for at least 6 days.

### DNA Purification

Colonic contents and mucosal scrapings were processed for DNA extraction using the Qiagen DNA Stool MiniKit following the manufacturer’s recommendations. Mucosal scrapings were then processed through the Qiagen PCR Purification Kit (Qiagen part 28106) following the manufacture’s recommendations to remove some residual extraction buffer salts that remained after the original purification. Following DNA purification, samples were screened for DNA concentration and purity using a Nanodrop DNA Flouremeter and the Qubit fluorometer (Life Technologies, Grand Island, NY, USA) and DNA was stored at −80°C prior to downstream processing.

### 16S Sequencing

DNA from the extracted colonic content samples and paired mucosal scrapings were submitted to Argonne National Laboratory—Institute for Genomics and Systems Biology Next Generation Sequencing Core (http://ngs.igsb.anl.gov/) for metagenomic analysis using the V4 region of the bacterial 16S rRNA gene. All samples were processed by the routine methodology of the core laboratory. Briefly, amplicons were synthesized using a universal 16S forward primer (515F) and individual unique Golay barcoded reverse primers (806R) as described (9). Appropriate positive and negative controls were included by the sequencing facility. Sample library DNA concentrations were quantified and samples were pooled with equal amounts of DNA. The pooled libraries were cleaned up with the MO-BIO UltraClean PCR Clean-Up Kit and the concentration was then diluted to 2 nM. For each sample type, a single flow cell lane containing 100 samples (the 36 samples of this report and 64 additional samples) of 300-bp paired end sequences was run on the Illumina MiSeq.

### Metagenomic Data Analysis

Forward and reverse reads from the paired end sequencing were first merged using the fastq.join script. Qiime 1.8 was then used for additional data analysis. De-multiplexing and quality filtering were then performed using the split_libraries_fastq.py script. The pick_reference_otus_through_otu_table.py script was used for operational taxonomic unit (OTU) calling and taxonomic assignment was performed based on the greengenes database (10). All libraries were adjusted to 47,000 reads for luminal contents and 56,000 reads for mucosal scrapings to avoid potential interpretation errors due to variable sampling depth. Comparisons of specific OTUs within groups were made at the phylum, order, and genus level and only those OTUs detected in at least 25% of samples were included in the analysis. Biological effect sizes were estimated using the linear discriminant analysis effect size (LEfSe) method (11) and a Procrustes analysis was performed to compare profiles generated from paired contents and scrapings in individual pigs (10). For colonic mucosal scraping samples, a CoVennTree (Comparative weighted Venn Tree) analysis (12) was performed to assess differences in the microbial population structure between pigs with SD and those inoculated pigs that did not develop SD.

### Statistical Analyses

Statistical output was generated by Qiime 1.8. Alpha diversity (chao1) was compared using a non-parametric two sample *t*-test with 999 Monte Carlo permutations. Beta diversity (Bray-Curtis dissimilarity) was compared using a two-sided student’s two-sample *t*-test with Bonferroni correction. The frequency of detection (group significance) of specific OTU calls within groups was compared using a Kruskal–Wallis non-parametric analysis of variance followed by correction for multiple comparison using the Benjamini and Hochberg False Discovery Rate (FDR) method (13). A FDR of 5% was utilized to determine significance. Firmicutes:Bacteroidetes ratios were calculated based upon the relative abundance percentages reported in Qiime and were compared using a two-sided student’s two-sample *t*-test. For outputs where *P-*values were derived, statistical significance was defined as *P* < 0.05.

## Results

**Figure 1:**
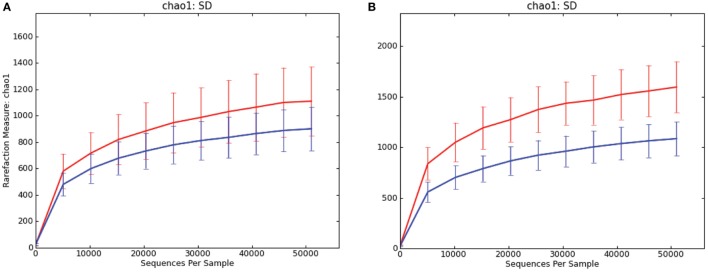
Rarefaction curves comparing alpha diversity (chao1) of microbiota samples from pigs with swine dysentery (blue lines) and without dysentery (red lines) after experimental inoculation. Samples of both colonic luminal contents **(A)** and colonic mucosal scrapings **(B)** reveal significant differences in richness between disease states (*P* = 0.014 and *P* = 0.001, respectively).

In both luminal content samples and mucosal scrapings, there were significant differences in richness (chao1; *P* = 0.014 and *P* = 0.001, respectively; Figure 1) and beta diversity (*P* < 0.001, both sample types) between samples from pigs with and without SD. The Procrustes analysis revealed generally similar spatial clustering by disease classification yet with a relatively high *M*^2^ value (0.44).

**Figure 2:**
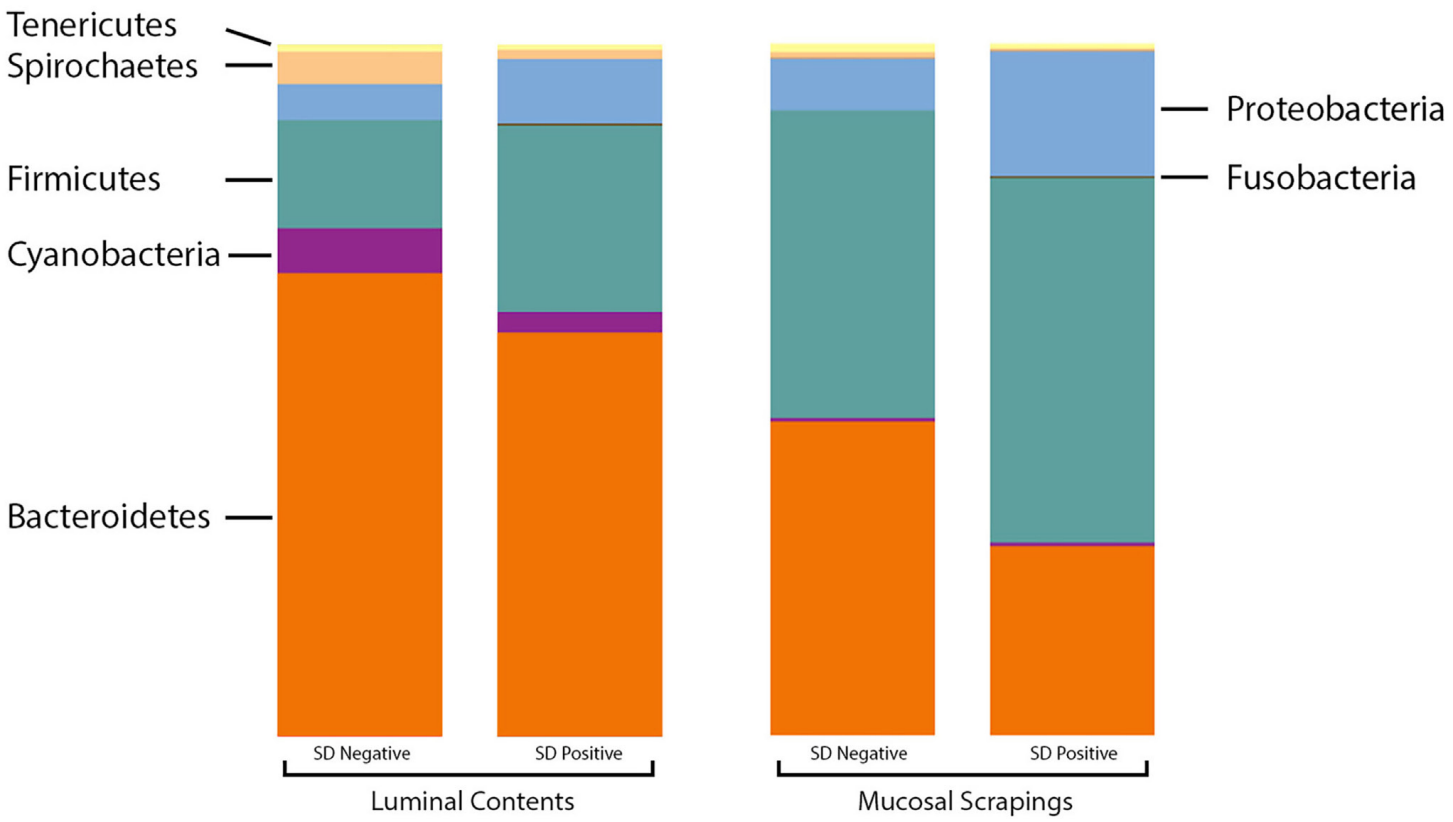
Stacked bar charts representing proportional abundance of major phyla in microbiota from 9-week-old pigs with and without swine dysentery (SD). The bars on the left reflect comparison of colonic luminal content samples and bars at the right refelct comparison of colonic mucosal scrapings. The *Firmicutes:Bacteroidetes* ratios in luminal content samples were significantly higher in pigs with SD relative to inoculated pigs that did not develop disease (*P* = 0.001).

At the phylum level, the relative abundance of *Firmicutes* was greater in the luminal samples of pigs with SD whereas *Proteobacteria* and *Fusobacteria* were more abundant in the mucosal scrapings of diseased pigs relative to those not developing disease (Figure 2). *Bacteroidetes* and *Synergistetes* were more abundant in scrapings from pigs that did not develop SD. The *Firmicutes:Bacteroidetes* ratios in luminal content samples were significantly higher in pigs with SD (mean 0.508 ± 0.226) relative to inoculated pigs that did not develop disease (mean 0.250 ± 0.134) (*P* = 0.001).

**Figure 3:**
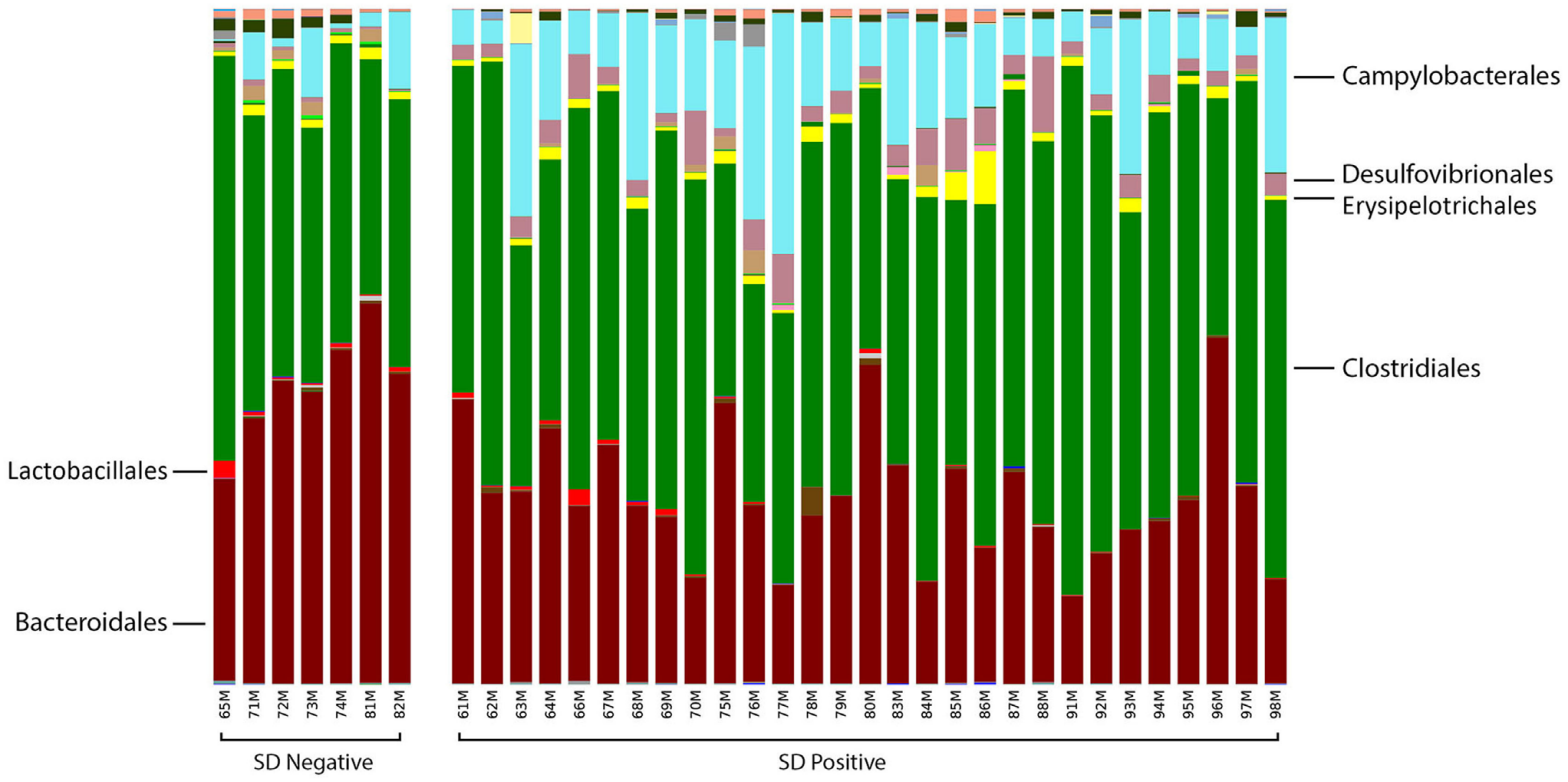
Stacked bar charts representing proportional abundance of major bacterial orders in the microbiota of colonic mucosal scrapings from 9-week-old pigs with or without swine dysentery following experiemntal inoculation with *Brachyspira hyodysenteriae* or *Brachyspira hampsonii*. Bars represent results of 16S rRNA gene sequencing followed by assignment of operational taxonomic units. Pigs that developed dysentery had an increased relative abundance of *Brachyspirales* (total percentage too small to be visible in this graph), *Campylobacterales*, and *Desulfovibrionales*, whereas those pigs that did not develop disease had increases in abundance of *Bacteroidales* and *Synergistales* (not visible in this graph). A false discovery rate of 5% was used to determine significance.

At the order level, the relative abundance of *Brachyspirales, Campylobacterales, Desulfovibrionales*, and *Enterobacteriales* was higher in pigs with SD for both mucosal scrapings (Figure 3) and luminal samples while *Clostridiales, Erysipelotrichales*, and *Fusobacteriales* were more abundant in the luminal contents only. For inoculated pigs that did not develop SD, *Burkholderiales* were more abundant in both sample types, *Bacteroidales* and *Synergistales* were more abundant in scrapings, and *Lactobacillales* and *Bifidobacteriales* were more abundant in luminal contents only.

**Figure 4:**
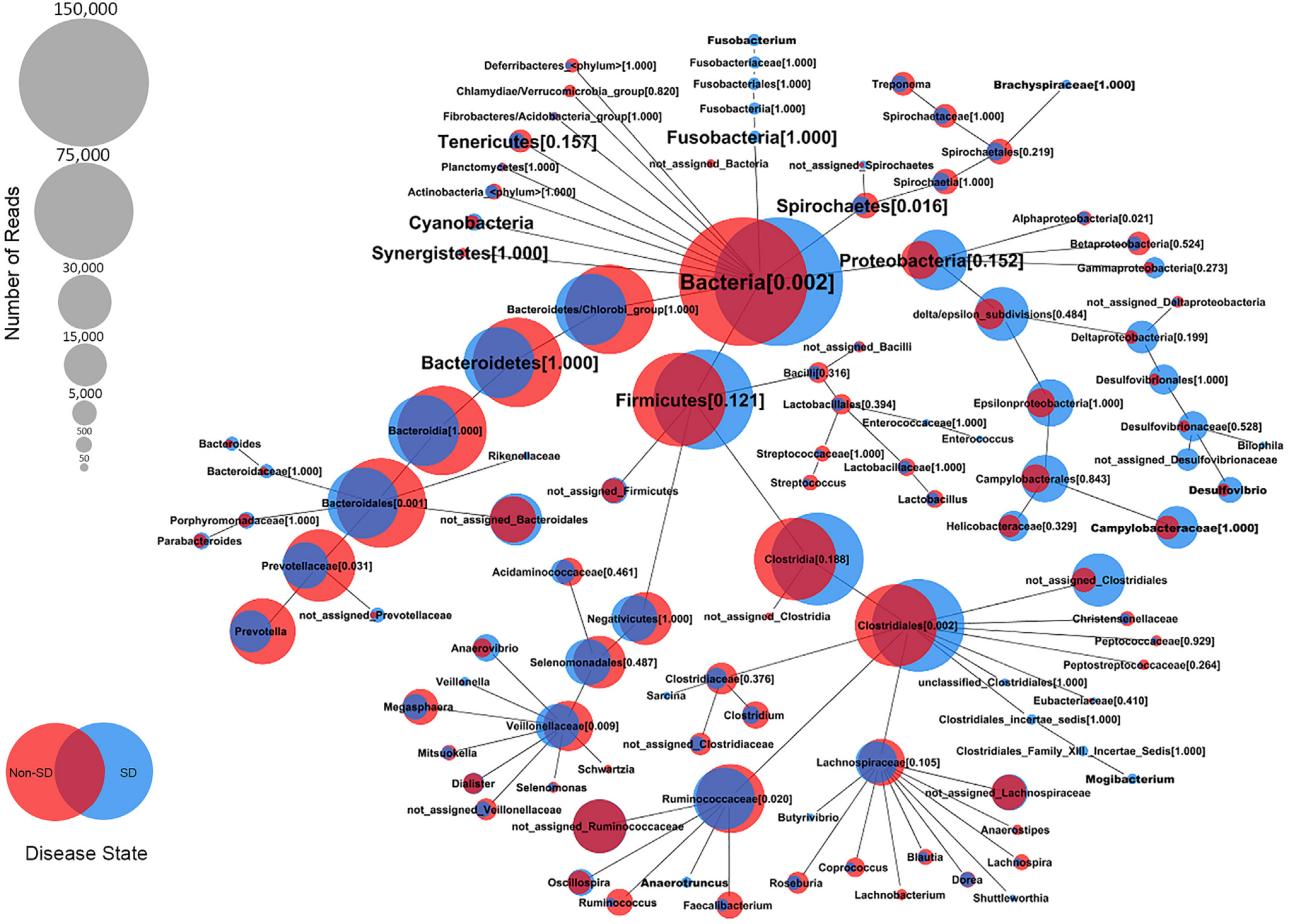
Comparative weighted Venn tree (CoVennTree) based on partial 16S rRNA gene sequences in the microbiota of colonic mucosal scrapings from pigs that did (blue circles) and did not (red circles) develop swine dysentery after experimental inoculation. The numbers in parentheses refer to Venn decomposition similarity (VDS value) and reflect the degree of similarity of all child nodes after the parent node (1 = identical). The overlap of weighted Venn circles of parental nodes reflects sequence reads originating from the same organism (group). Libraries were normalized to 150,000 reads and singletons were excluded.

At the genus level, the relative abundance of *Brachyspira, Campylobacter, Fusobacterium*, and *Mogibacterium* was higher in pigs with SD for both mucosal scrapings and luminal samples while *Desulfovibrio* were more abundant in scrapings only and *Flexispira* were more abundant in the luminal contents only. For inoculated pigs that did not develop SD, *Streptococcus* and *Ruminococcus* were more abundant in both sample types while *Prevotella* and *Roseburia* were more abundant in scrapings only. *Brachyspira* OTUs were detected in 22 of 29 luminal content samples and 29 of 29 mucosal scrapings from pigs with SD versus none (0 of 7) of the luminal content samples and only one mucosal scraping sample from inoculated pigs that did not develop SD. When detected, the relative abundance of *Brachyspira* was low averaging 0.02% in mucosal scrapings and 0.006% in luminal content samples. A CoVennTree analysis of the microbiota in mucosal scraping samples from pigs with and without SD reveals the relative size (number of reads) and similarity (degree of overlap) of detected taxa (Figure 4).

**Figure 5:**
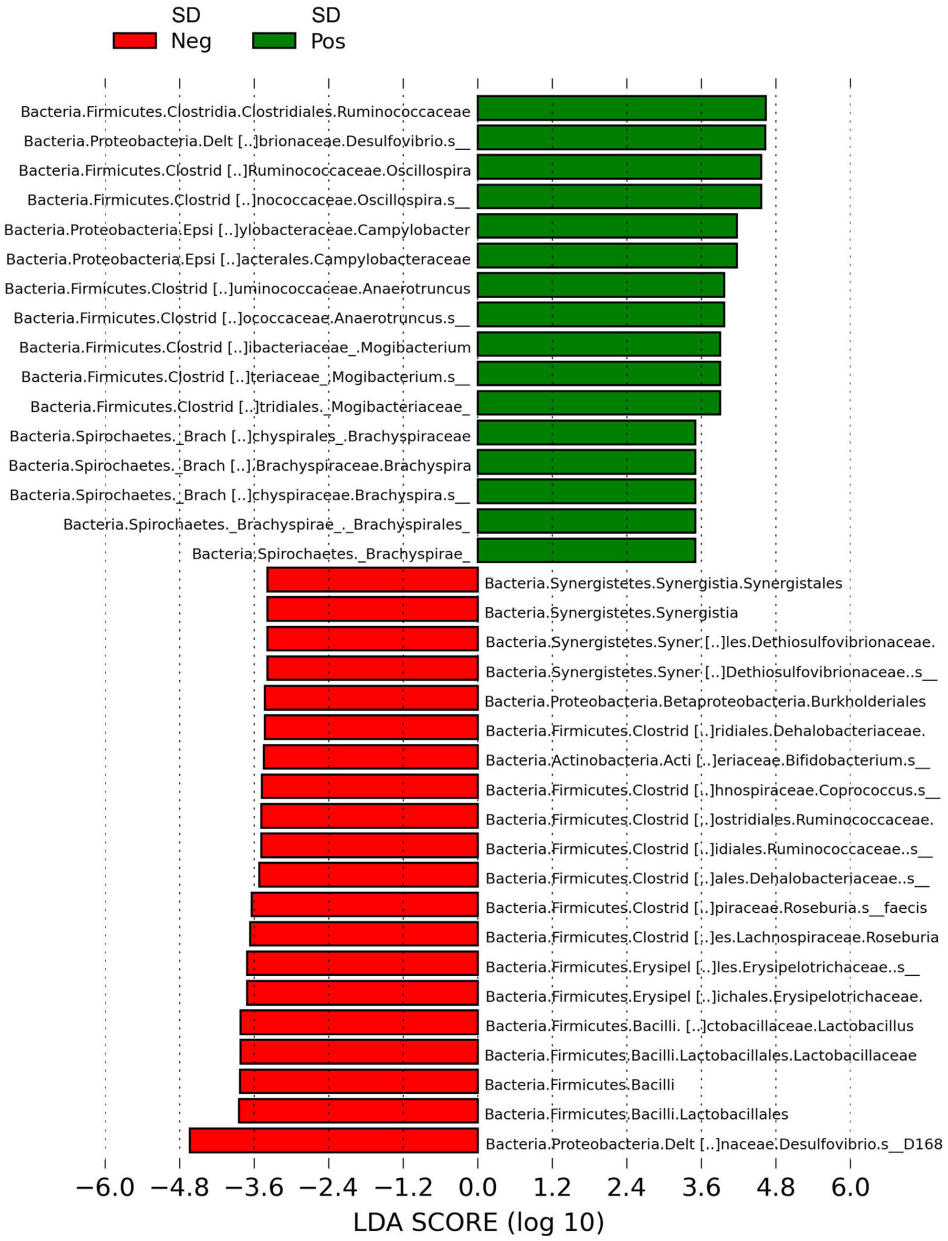
Histogram of linear discriminant analysis (LDA) scores computed by LEfSe revealing differentially abundant taxa in the microbiota of mucosal scrapings from pigs with or without swine dysentery (SD) following experimental inoculation with *Brachyspira hyodysenteriae* or *Brachyspira hampsonii*. *Brachyspira, Campylobacter, Mogibacterium, Anaerotruncus, Oscillospira*, and multiple *Desulfovibrio* spp. were differential features in mucosal scrapings from pigs with SD while *Lactobacillus, Roseburia, Synergistales*, a *Bifidobacterium* spp., and a specific *Desulfovibrio* spp. were characteristic of samples from those pigs that were resistant to infection and disease development.

**Figure 6:**
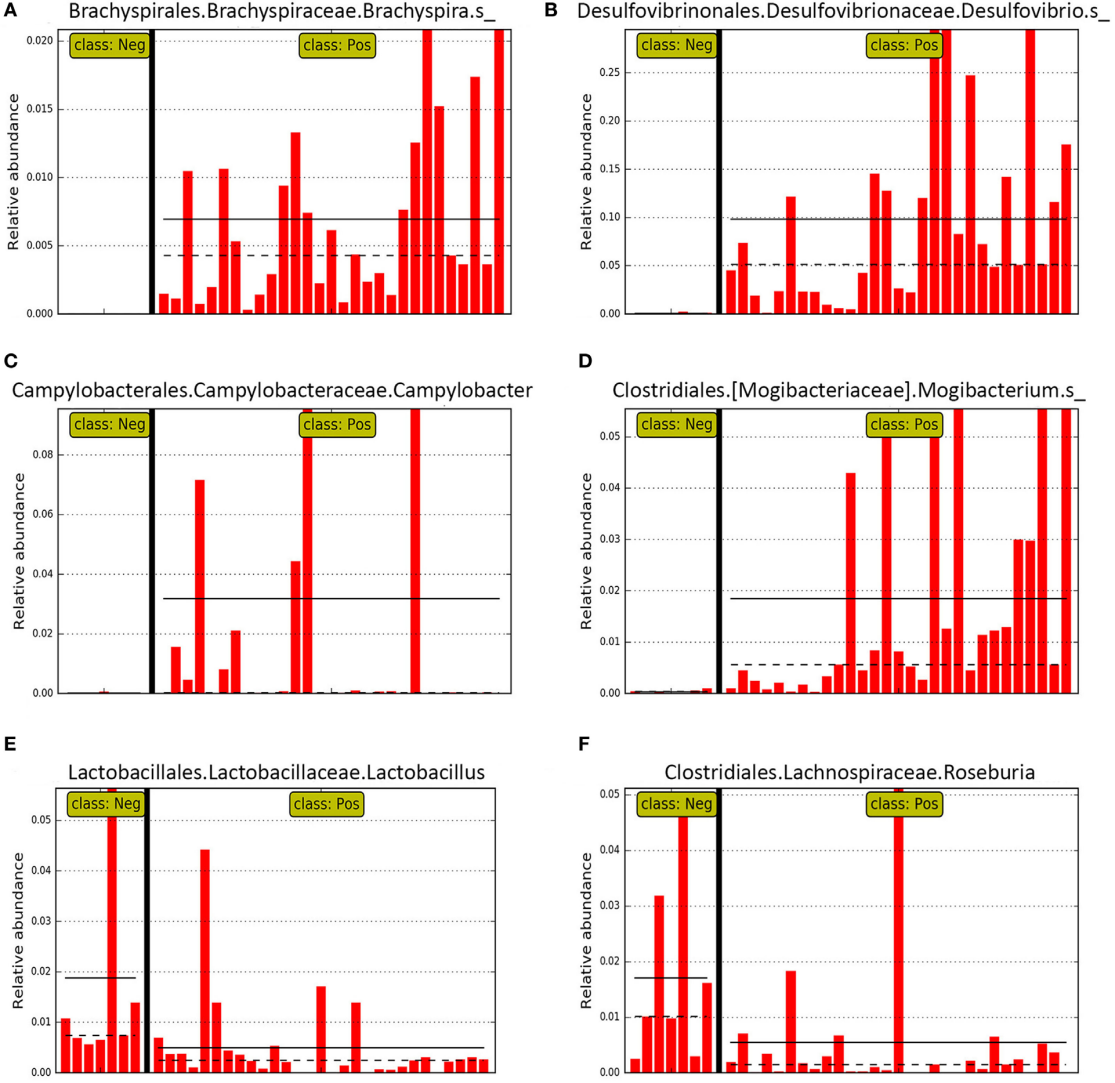
Bar graphs representing relative abundance in individual pig mucosal scraping samples of specific genera identified as differential features in LEfSE for pigs with (class: Pos) and without (class: Neg) swine dysentery (SD) after experimental inoculation. Solid black horizontal lines represent the mean abundance within each class. Panels **(A–D)** represent genera that were differential features of the microbiota from pigs with SD, whereas panels **(E,F)** represent two genera that were differential for the mucosal microbiota from pigs without SD.

LEfSe revealed *Brachyspira, Campylobacter, Mogibacterium, Oscillospira, Anaerotruncus*, and multiple *Desulfovibrio* spp. as differential features in mucosal scrapings from pigs with SD while *Lactobacillus, Roseburia, Synergistales*, a *Bifidobacterium* spp., and a specific *Desulfovibrio* spp. were differential in pigs without disease (Figure 5). The Plot One feature of LEfSe provides a more detailed comparison between samples (Figure 6).

The *Brachyspira* OTUs detected were a 100% match over the region compared to *B. hyodysenteriae* and *B. hampsonii*. The predominant *Campylobacter* OTU detected had high nucleotide sequence homology (100%) based on a blast search to *Campylobacter hyointestinalis* and the predominant *Desulfovibrio* OTUs detected in SD pigs had high nucleotide sequence homology (95%) based on a blast search to *Desulfovibrio desulfuricans*. One specific *Desulfovibrio* OTU was a differential feature in LEfSe for pigs without SD, and this OTU had high nucleotide sequence homology (99%) based on a blast search to *Desulfovibrio* spp. Marseille-P2429.

## Discussion

While Procrustes analysis of the microbiota revealed similar clustering of samples by disease status, the *M*^2^ value suggests the data sets are not a particularly good fit and that differences exist between the microbiota of the colonic contents and scrapings from the same pig. This is consistent with a recent report where microbial profiles from the cecal mucosa and cecal content of pigs differed significantly with luminal content samples being more diverse (14). While not unexpected, this paired analysis emphasizes that exploration of both sample types may be warranted when studying specific intestinal diseases and exploration of the mucosal microbiome specifically may yield more specific information regarding those species in more intimate contact with the host.

Significantly higher *Firmicutes:Bacteroidetes* ratios were observed in the luminal contents of pigs with SD relative to those without disease, which is consistent with previous studies where higher ratios have been reported in both dogs and humans with diarrhea regardless of cause (15, 16). Altered ratios were also reported in the fecal microbiota from pigs with SD after inoculation with *B. hampsonii* (17); however, in this previous investigation, there were no statistically significant differences detected in the microbiota of feces from pigs that did and did not develop mucohemorrhagic diarrhea. Possible explanations for the discord between this previous investigation and the current study include sequencing depth (samples were normalized to 1,000 reads in the previous investigation versus 47,000 and 56,000 in the current study), examination of feces versus luminal contents and mucosal scrapings, limitation to phylum level analysis in the previous report versus order and genus level herein, and the inclusion of sham-inoculated controls in the previous investigation as it is unknown if the profiles from those pigs would have been susceptible or resistant to disease expression.

In the study of this report, both luminal contents and mucosal scrapings from inoculated pigs with SD had significant differences in the relative abundance of multiple bacteria at the order level when compared with samples from inoculated pigs that did not develop disease. Previous investigations have revealed that when specific anaerobes, such *Fusobacterium necrophorum*, are present in the microbiota, pigs are rendered susceptible to SD development following inoculation with *B. hyodysenteriae* (3). Consistent with this finding, *Fusobacteria* were more abundant in luminal contents of pigs that developed SD after inoculation. A significant increase in *Fusobacterium* has also been reported in fecal samples from weaned pigs with diarrhea associated with porcine epidemic diarrhea virus infection (18) as well as in nursing pigs with non-specific diarrhea (19) suggesting that this finding may also be reflective of dysbiosis associated with certain types of diarrhea.

*Campylobacter* (*Vibrio*) *coli* has been historically associated with pigs with SD and at one point was considered a potential etiologic agent prior to the identification of *Treponema hyodysenteriae* (now *B. hyodysenteriae*) (20). In an investigation of fecal samples from pigs with diarrhea of various causes, a positive *Campylobacter* culture was obtained from all pigs with a laboratory diagnosis of *Brachyspira*-associated disease suggesting a potential interrelationship between these bacteria in the large intestine of pigs (21). Not surprisingly, this association is clearly supported by the data of the current investigation where *Campylobacterales* were more abundant in all samples types from pigs with SD. *Campylobacter* spp. are commonly present in the mucosa-associated microbiota of the porcine large intestine (14), and their enrichment in SD may also reflect perturbation of the normal mucosal community after colonization of this niche by *Brachyspira*.

LEfSe further revealed that both *Brachyspira* and *Desulfovibrio* spp. were differential features at the mucosal surface of pigs with SD, which is of note as *Desulfovibrio* spp. are sulfate reducing bacteria with the potential to degrade the sulfated mucins that comprise part of the mucus barrier and utilize mucin as a substrate (22). Indeed, a reduction in sulfated mucins has been previously reported in pigs with acute SD (23) and this breakdown in the organization of colonic mucus has been shown to provide more mucin-binding sites for *B. hyodysenteriae* (24). *Desulfovibrio* spp. are also consistently increased in colonic biopsies from people with ulcerative colitis (25) and it is proposed that these bacteria may contribute to colitis through the production of toxic hydrogen sulfide, a byproduct of metabolism of sulfated mucins (26). *D. desulfuricans* has increased affinity to mucins from patients with ulcerative colitis; however, strain-specific differences in mucin binding capacity have been observed with some strains having reduced affinity relative to the type strain (22). This is consistent with the findings of the present report where a majority of detected *Desulfovibrio* OTUs were differential for scrapings from pigs with SD while a single OTU was differential for pigs that did not develop SD. *Brachyspira* accounted for an extremely small proportion of the total microbiota (≤0.02%), even in mucosal scrapings from pigs with acute SD.

*Mogibacterium, Anaerotruncus*, and *Oscillospira* were also differential features of the microbiota from pigs with SD; however, little has been reported regarding the role of these bacteria in animals. *Mogibacterium* is an oral bacterium commonly associated with human periodontal disease and has been shown to have greater relative abundance in the fecal microbiota of people with adenomatous polyps versus those without (27). *Anaerotruncus* is a recently described organism that has been detected in human feces and a single case of human bacteremia (28). *Oscillospira*, on the other hand, is an anaerobic bacterium that is reduced in several human inflammatory conditions (29) and thus its association with SD seems contradictory. Further investigation into the role of these bacteria in health and disease in livestock species is needed.

*Lactobacillus* and a *Bifidobacterium* spp., two genera commonly investigated as probiotic agents, were differential features in the mucosa of inoculated pigs that did not develop SD. These and other lactic acid-producing bacteria can reduce luminal pH, produce bacteriocins, and protect biological niches through competitive exclusion. *Lactobacillus* spp. exhibit significant differences in their ability to bind mucin and to exclude *Salmonella* Typhimurium and *Escherichia coli in vitro*, suggesting certain species may be more beneficial in reducing pathogen binding *in vivo* (30). A significant reduction in *Lactobacillus* spp. was observed in pigs consuming 30% DDGS relative to pigs fed a standard corn-soy diet (4), and pigs consuming this same diet (30% DDGS) had a shorter time to onset of SD following *B. hyodysenteriae* infection relative to controls (8) further suggesting decreased *Lactobacillus* may be one potential biomarker of susceptibility to SD development. Reductions in *Lactobacillus* have also been reported in nursing pigs with diarrhea of unspecified cause (19). Lactobacilli and bifidobacteria produce lactate, which in turn can be used by certain butyrate-producing bacteria, such as *Megasphaera* (6), and increased levels of this short chain fatty acid may improve colonic health.

Butyrate is the primary energy source for colonocytes and has also been shown to modulate the immune system, suppress cancer, reduce oxidative stress, and modulate intestinal motility modulation (31). In the current study, *Roseburia*, a butyrate-producing anaerobe commonly detected in the pig colon (14), was enriched in mucosal scrapings of inoculated pigs that did not develop SD, and *Roseburia faecis* specifically was a differential feature of the microbiota from pigs without disease. This is consistent with previous reports suggesting *Roseburia* as a biomarker of a healthy gut (32).

In summary, the microbial profiles of mucosal scrapings were different than those in adjacent luminal contents but reveal similar clustering by disease phenotype. Mucosal scraping samples offered better detection of agents of SD and are likely a more direct assessment of the ecological niche of SD. Evaluation of the microbiota in mucosal scrapings revealed numerous potential biomarkers of disease (*Desulfovibrio, Campylobacter, Mogibacterium*, and *Fusobacterium*) and absence of disease development (*Lactobacillus, Bifidobacterium*, and *Roseburia*) following inoculation with agents of SD; however, it remains to be determined if these differences are a consequence of SD or related to cause. Investigation into the potential role of these bacteria in the pathogenesis of SD or in resistance to disease expression and thereby the utility of their detection in the microbiota in evaluating potential prebiotic approaches to SD control is warranted.

## Ethics Statement

All animal procedures were approved by the Institutional Animal Care and Use Committee of Iowa State University (Log Number: 1-12-7283).

## Author Contributions

Conceived and designed the experiments, analyzed the data, and contributed reagents/materials/analysis tools: EB and PP. Performed the experiments: PP and BA. Wrote the paper: EB.

## Conflict of Interest Statement

The authors declare that the research was conducted in the absence of any commercial or financial relationships that could be construed as a potential conflict of interest.
